# Optical, structural and antibacterial properties of phase heterostructured Fe_2_O_3_–CuO–CuFe_2_O_4_ nanocomposite

**DOI:** 10.1038/s41598-024-64090-9

**Published:** 2024-06-22

**Authors:** Adnan Alnehia, Muhammad Hadi, Hisham Alnahari, Annas Al-Sharabi

**Affiliations:** 1 Department of Physics, Faculty of Applied Sciences, Thamar University, 87246 Dhamar, Yemen; 2 Department of Physics, Faculty of Sciences, King Khalid University, P.O.Box 9004, Abha, Saudi Arabia; 3 Department of Physics, Faculty of Sciences, Sana’a University, 12081 Sana’a, Yemen

**Keywords:** Materials science, Nanoscience and technology

## Abstract

The synthesis of the Fe_2_O_3_–CuO–CuFe_2_O_4_ nanocomposite was effectively achieved through the sol–gel technique, utilizing ethanol as a reactive fuel. Investigation of the nanocomposite’s structure via X-ray Diffraction confirmed the coexistence of Fe_2_O_3_, CuO, and CuFe_2_O_4_ phases within the material. The Scherrer equation was applied to determine an average crystallite size ranging from 60 to 95 nm. UV–visible spectroscopy studies suggested the material possesses an approximate energy bandgap of 4 eV. Scanning Electron Microscopy provided insights into the nanocomposite’s surface morphology, which exhibited a porous and heterogeneous aggregation of particles in various sizes and shapes. When tested for antibacterial efficacy, the nanocomposite exhibited activity against gram-positive *S. aureus* with a maximum zone of inhibition (ZOI) measuring 9 mm at the highest concentration, whereas no inhibitory effect was detected against gram-negative *E.coli*.

## Introduction

Metal oxide nanocomposites (NCs) have garnered significant attention in recent years due to their unique properties, including optical, electrical, mechanical, photocatalytic, thermal, and structural characteristics^1–5^. These NCs, formed by combining two, three, or more oxides at the nanometer scale, find applications in photovoltaic devices, solar cells, battery materials, UV detectors, gas sensors, and fuel cells, among others^6–11^. The combination of various metal oxides into a new nanocomposite enhances the individual properties of the oxides and opens up new avenues of research for biological, optoelectronic, thermal, and electrical applications^7,12–14^. Introducing a new phase to a composite material can alter its electronic properties. Metal oxide nanocomposites are prepared using various methods such as hydrothermal synthesis^15^, co-precipitation^16^, self-combustion^17^, ultrasonic-assisted routes^18^, and the sol–gel method ^19,20^. The sol–gel method, in particular, is preferred for its low cost, speed, and simplicity, requiring no expensive equipment. CuO, a p-type semiconductor with an optical band gap of 1.22 eV, exhibits excellent optical, electrical, and thermal properties and is used in diverse applications such as supercapacitors, superconductors, catalysis, gas sensing, and photocatalytic activity. Additionally, it is non-toxic and economically feasible ^21–27^. Fe_2_O_3_, an n-type semiconductor with an energy band gap of 2.1 eV, possesses chemical stability and natural abundance. It is utilized in various applications while also being non-toxic and cost-effective^28–30^. Globally, bacterial diseases pose a significant threat to human health, with foodborne pathogens like *Staphylococcus aureus* (*S. aureus*) and *Escherichia coli* (*E. coli*) causing serious illnesses^31^. Furthermore, antibiotic resistance in these bacteria has become a major concern. To combat this, the development of new inorganic antibacterial agents is crucial. Metal oxide NCs have shown promise in this regard due to their large surface areas and ability to generate charged radicals that hinder bacterial growth^32–34^.

In this study, we synthesized a mixed oxide of copper and iron using the sol–gel method, which has been proven to be an efficient and cost-effective route for producing complex oxide materials. Our XRD results confirmed the formation of CuO–Fe_2_O_3_–CuFe_2_O_4_, a promising material for various applications such as catalysis, sensors, medicine, and energy storage. Complementing these findings, UV–visible spectroscopy analysis revealed an energy bandgap of 4 eV, further underscoring the material’s potential for optoelectronic applications.The novelty of this work lies in the successful synthesis of these mixed oxides with controlled composition and phase purity, showcasing the potential of our approach for the development of advanced functional materials.

## Materials, method and characterization techniques

### Materials

Copper nitrate Cu(NO_3_)_2_.3(H_2_O) (BDH, 97%), iron nitrate Fe(NO_3_)_3_.9(H_2_O) (Sigma–Aldrich, 98%) and ethanol (Eth)were used without further purification. The test bacteria* Escherichia coli* (*E. coli*) and *Staphylococcus aureus* (*S. aureus*) were generously provided by Alfa Medical Laboratory in Dhamar city, Yemen. Mueller–Hinton Agar (MHA) was obtained from Sigma-Aldrich in Darmstadt, Germany.

### Preparation of the sample

The Fe_2_O_3_–CuO–CuFe_2_O_4_ nanocomposite (NC) was synthesized using the sol–gel method^20,35–38^. Initially, two separate solutions were prepared: one with Cu(NO_3_)_2_·3H_2_O (5.798 g in 30 mL of ethanol) and another with Fe(NO_3_)_3_·9H_2_O (9.69 g in 30 mL of ethanol), keeping a molar ratio of 1:1. Each solution was stirred for fifteen minutes at a temperature of 22 ± 2 °C until homogeneity was achieved. These solutions were then combined and stirred continuously at 80 °C for seventy minutes. During this time, the mixture gradually dried while being heated and mixed, eventually forming a gel that turned into a dry mass. Subsequently, this dry mass was ground into a fine powder and annealed at a temperature of 800 °C for 120 min. After completing these procedures, the composite was ready for subsequent characterization.

### Antibacterial test

The antibacterial effectiveness of Fe_2_O_3_–CuO–CuFe_2_O_4_ NC was evaluated in vitro using the widely recognized disk diffusion method^39–41^. The assessment involved testing against specific Gram-positive (*S. aureus*) and Gram-negative (*E. coli*) bacterial strains, with azithromycin (AzM) serving as the reference standard^35^. Initially, the bacterial strains were cultured at 37 ± 1°C for 24 h and then adjusted with nutrient broth to achieve an absorbance of 0.075–0.1. Test plates were prepared by spreading the bacteria over MHA media in Petri dishes. Subsequently, 6 mm diameter disks, each wetted with 20μL on both sides containing various concentrations (50, 100, and 200 mg/mL) of Fe_2_O_3_–CuO–CuFe_2_O_4_ NC, were placed on the media surface and incubated at 37 ± 1 °C for 20–23 h. The antibacterial activity was assessed by measuring the diameter of the inhibition zone (ZOI) using a ruler and comparing the results with the controls^5^. The experiment was conducted in duplicate, and the average values were reported.

### Characterization techniques

The structural properties were analyzed using X-Ray Diffraction (XRD) with a XD-2 X-ray diffractometer employing Cu Kα radiation (λ = 1.54Å), while the morphological properties were examined using SEM (Jeol Ltd., Tokyo, Japan). The optical properties were studied through UV–Vis spectroscopy using a Hitachi U3900 instrument with Varian Cary 50 software.

## Results and discussions

### X-ray diffraction

**Figure 1:**
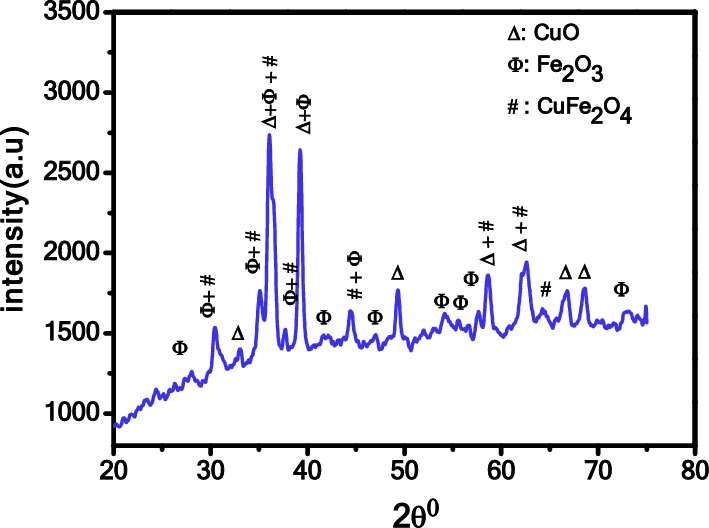
XRD pattern of the Fe_2_O_3_–CuO–CuFe_2_O_4_ nanocomposite.

The X-ray diffraction (XRD) pattern shown in Fig. 1 indicates the formation of Fe_2_O_3_–CuO–CuFe_2_O_4_ nanocomposite after being prepared and annealed at 800 °C. The diffraction peaks confirm the presence of cubic Fe_2_O_3_, monoclinic CuO, and tetragonal CuFe_2_O_4_ structures, with no impurities or secondary phases present. The specific diffraction peaks and reflection planes for Fe_2_O_3_, CuO, and CuFe_2_O_4_ are consistent with JCPDS card No. 39-1346, 45-0937, and 34-0425, respectively^6,9,19,42^.The average crystallite size($D$) of the Fe_2_O_3_–CuO–CuFe_2_O_4_ nanocomposite was determined using Scherer’s formula (Eq. 1)^43,44^, with the calculated size recorded in Table 1.

1 $$D = \frac{0.9\lambda }{{\beta cos\theta }}$$

where λ is the XRD wavelength (1.5406 A), θ is the Bragg diffraction peak (in radian), and β is the full width at half maximum (FWHM). Additionally, the dislocation density (δ) was computed using Eq. (2), providing information about vacancies and defects in the crystal. Also, the lattice strain ($\varepsilon$) of nanocomposite $\varepsilon =\beta cos\theta /4$ was computed. The variation in the strain is may be due to the alteration in structure, crystallite size and morphology. The estimated dislocation density and the strain values are also recorded in Table 2^35,45,46.^

2 $${\updelta } = \frac{1}{{D^{2} }}$$

**Table 1:** The crystallite size, 2-theta, FWHM, d-spacing, strain and average dislocation density of Fe_2_O_3_, CuO, and CuFe_2_O_4_ within the prepared Fe_2_O_3_–CuO–CuFe_2_O_4_ nanocomposite, as determined through X-ray diffraction (XRD) analysis.

Oxides	2-theta (2θ^0^)	(hkl)	FWHM (degree)	d-spacing (Å)	Crystallite size(nm)	average D(nm)	average dislocation density(lines/m^2^)*10^14^	The strain (*ε*)
Fe_2_O_3_	25.923	211	0.194	3.434	42.024	61.102	2.68	0.000825
	34.968	310	45.269	2.632	0.184			0.000765
	44.736	410	45.941	2.024	0.187			0.000754
	59.437	520	128.83	1.553	0.071			0.000269
	62.839	440	35.529	1.477	0.262			0.000975
	64.368	441	69.015	1.452	0.136			0.000502
CuO	39.240	200	26.032	2.294	0.324	96.161	1.08	0.001331
	58.365	202	124.64	1.579	0.073			0.000278
	66.448	− 311	64.161	1.406	0.148			0.000540
	72.485	311	169.80	1.303	0.058			0.000204
CuFe_2_O_4_	29.730	112	124.54	3.003	0.066	73.831	1.83	0.001366
	34.968	103	45.269	2.632	0.184			0.000304
	36.001	211	29.414	2.493	0.284			0.000614
	41.832	004	74.606	2.157	0.114			0.000236
	52.813	204	140.79	1.732	0.063			0.001024
	62.124	224	28.359	1.493	0.327			0.000508

**Table 2:** The lattice constants, atom number per unit cell (Z) and volume of Fe_2_O_3_, CuO, and CuFe_2_O_4_ within the synthesized Fe_2_O_3_–CuO–CuFe_2_O_4_ nanocomposite.

Oxides	a (Å)	b (Å)	c (Å)	z	Volume (Å^3^)	Density (g/cm^3^)
Fe_2_O_3_	8.356	8.356	8.356	10.67	583.4	4.9
CuO	4.685	3.426	5.130	4	81.2	6.45
CuFe_2_O_4_	5.844	5.844	8.630	4	294.8	5.39

Lattice strain often arises due to discrepancies between the ideal and the actual distances of atoms within the crystal lattice, which can result from various synthesis conditions such as temperature, pressure, and the rate of formation. It is a critical parameter that provides insight into the defect density and crystallite size of the material. The lattice strain values suggest the presence of intrinsic stress within the nanocomposite, possibly induced by the heterogeneity of the constituent phases and their interfacial interactions. This strain can be beneficial or detrimental to the material’s properties, depending on the specific application. For instance, in certain cases, a higher lattice strain can lead to improved electrical or ionic conductivity due to the creation of more active sites, which is advantageous for catalytic applications or in energy storage devices like batteries and supercapacitors. Conversely, excessive lattice strain might compromise the structural integrity and decrease the mechanical stability of the material. Therefore, understanding the extent and the nature of lattice strain is essential for tailoring the synthesis process to enhance the desired properties of the nanocomposite for specific applications.

The lattice constants (a,b & c), unit cell volume (v), atom number per unit cell(Z), and the mass density(ρ) for the cubic Fe_2_O_3_, monoclinic CuO, and tetragonal CuFe_2_O_4_ structures were computed and recorded in Table 2^46,47^.

### SEM

**Figure 2:**
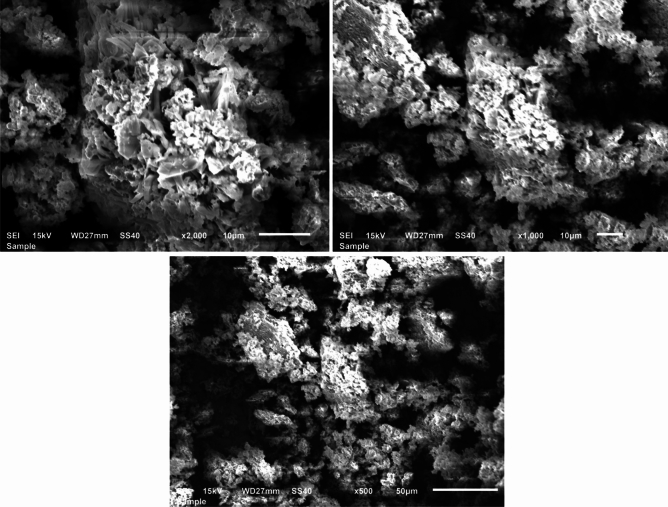
SEM image of the Fe_2_O_3_–CuO–CuFe_2_O_4_ nanocomposite.

The Scanning Electron Microscopy (SEM) analysis was performed to observe and understand the morphology of the prepared sample. Figure 2 displays the SEM images of the synthesized nanocomposite at different magnifications. Analysis of Fig. 2 confirms the presence of Fe_2_O_3_–CuO–CuFe_2_O_4_ nanocomposite, revealing numerous porous agglomerates with irregular sizes and shapes.

### UV–visible

**Figure 3:**
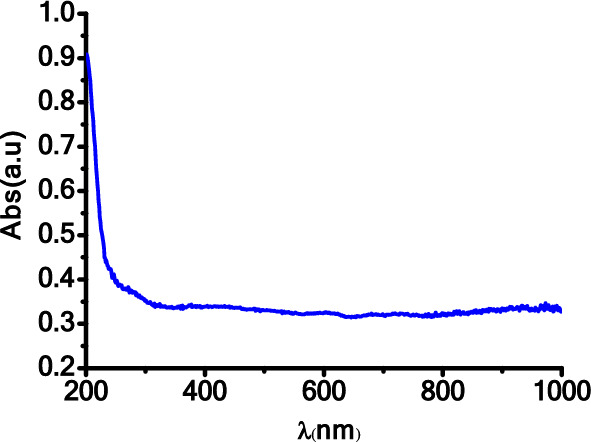
Absorbance spectra of the Fe_2_O_3_–CuO–CuFe_2_O_4_ nanocomposite.

**Figure 4:**
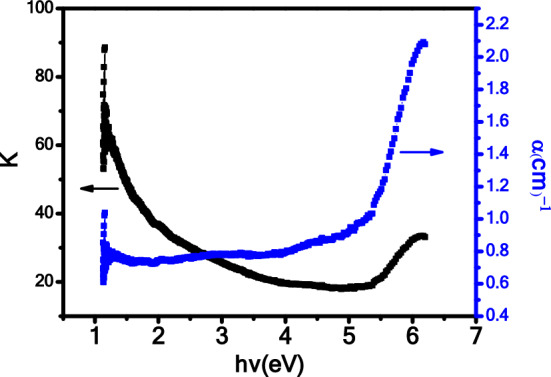
The absorption coefficient (α) and the extinction coefficient (k) of Fe_2_O_3_–CuO–CuFe_2_O_4_ nanocomposite.

**Figure 5:**
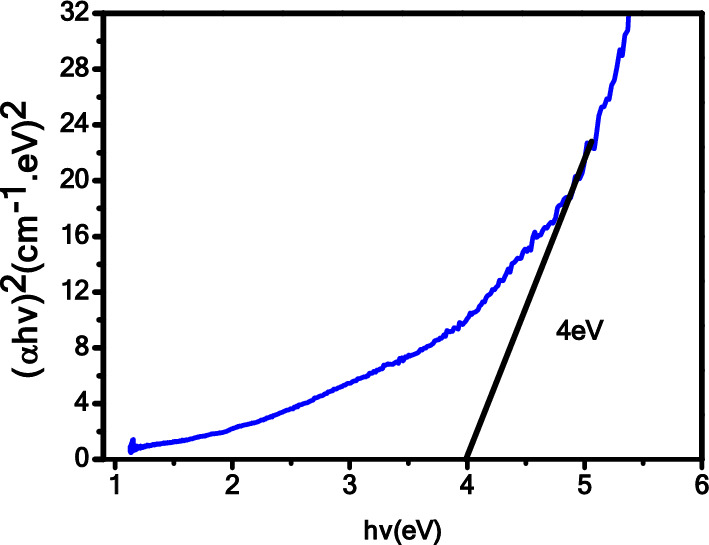
Optical band gap of Fe_2_O_3_–CuO–CuFe_2_O_4_ nanocomposite.

UV–Visible spectroscopy was utilized to investigate the optical properties of the Fe_2_O_3_–CuO–CuFe_2_O_4_ nanocomposite. In Fig. 3, the absorption spectra of the nanocomposite cover the range of 200–1000 nm, indicating that absorption was most prominent at shorter wavelengths and decreased gradually as the wavelength increased. Moreover, in Fig. 3, the UV–Vis absorbance spectrum of the synthesized CuO-Fe_2_O_3_-CuFe_2_O_4_ nanocomposite does not exhibit distinct strong absorption peaks, which is characteristic of the nature of the materials studied. The spectrum shows a significant absorption edge at approximately 290 nm, indicative of the bandgap of the composite. The absence of additional strong absorption peaks can be explained by the following: (i) Homogeneity of the Composite: The sol–gel method used for synthesis provides a uniform material with a consistent phase composition, which leads to broad absorption rather than sharp, localized peaks.(ii) Phase Interactions: The presence of multiple oxides within the composite can lead to interaction between the phases, which may result in a broadening of the absorption features, smoothing out any peaks that could be associated with individual oxide components. (iii) Particle Size Effects: The nanoscale size of the crystallites, ranging from 60 to 95 nm, can influence the optical properties of the composite. Nanoparticles can exhibit size-dependent absorption properties due to quantum confinement effects, which can lead to a shift in the absorption edge and alter the expected absorption peak profiles. (iv) Direct Bandgap Transitions: The synthesized composite possesses a direct bandgap, as evidenced by the sharp absorption edge, and does not typically show the sharp peaks that are characteristic of indirect bandgap transitions or d-d transitions often found in other types of metal oxide materials. (v) Surface States: The sol–gel synthesis and subsequent calcination at 800 °C may create a nanocomposite with fewer surface states that could give rise to distinct absorption peaks. Instead, a continuous distribution of states at the band edge results in a smooth absorption spectrum.The absorbance continuously decreases beyond the absorption edge, from 290 nm onwards up to 1000 nm, without showing any additional peaks, which is in agreement with the absorption behavior expected for nanocomposites with the aforementioned characteristics. The spectral profile obtained is consistent with optical transitions and bandgap absorption, and the data supports the composition and structure of the prepared nanocomposite as verified by XRD analysis. Additionally, Fig. 4 illustrates the changes in the absorption coefficient (α) and the extinction coefficient (k) of Fe_2_O_3_–CuO–CuFe_2_O_4_ with respect to wavelength. The data shows that the absorption coefficient (α) increases with the wavelength, while the extinction coefficient(k) is highest at shorter wavelengths and decreases as the wavelength increases. Tauc’s relation^48,49^ was employed to calculate the optical energy band gap, resulting in a value of 4 eV for the Fe_2_O_3_–CuO–CuFe_2_O_4_ nanocomposite, as shown in Fig. 5. The energy bandgap of approximately 4 eV in the synthesized CuO–Fe_2_O_3_–CuFe_2_O_4_ nanocomposite presents significant implications for its use across various applications. This value, indicative of the material’s ability to absorb UV photons, positions the nanocomposite as a promising candidate for photocatalytic activities which could include the degradation of organic pollutants and the production of hydrogen through water splitting. Additionally, the bandgap situated in the visible to UV spectrum enhances the material’s suitability for electronic and optical devices, such as UV photodetectors and the active layers of light-emitting diodes that operate within the blue/UV range. The semiconductor properties inherent to a bandgap of this magnitude suggest potential usage in high-power and high-frequency electronic devices, benefiting from the material’s good insulation characteristics. Furthermore, a larger bandgap typically confers greater chemical and thermal stability to the material, making it ideal for deployment in environments that may subject devices to extreme conditions. Therefore, optimizing the synthesis process to fine-tune the bandgap is crucial, especially as the interactions among the CuO, Fe_2_O_3_, and CuFe_2_O_4_ phases within the nanocomposite contribute to its distinctive electronic properties and the resulting bandgap observed^25,50^. The energy gap value calculated for this nanocomposite aligns with the results obtained by Kaveh Parvanak Boroujeni et al. from their study on the Fe_2_O_3_/CuO nanocomposite, which demonstrated an energy gap of 4.2 eV^50^.The observed 4 eV energy band gap for the Fe_2_O_3_–CuO–CuFe_2_O_4_ nanocomposite, determined using the sol–gel method, likely arises from the intricate interactions and interfaces between its constituent elements. When these materials are combined in a nanocomposite, potential synergistic effects may result from chemical reactions and interfacial interactions among the components. These interactions can lead to modifications in the energy band gap of the nanocomposite, which may not simply be a summation of the band gaps of the individual components due to the emergence of new energy states and the influence of interfacial interactions and structural changes at the nanoscale.

## Antibacterial activity

**Figure 6:**
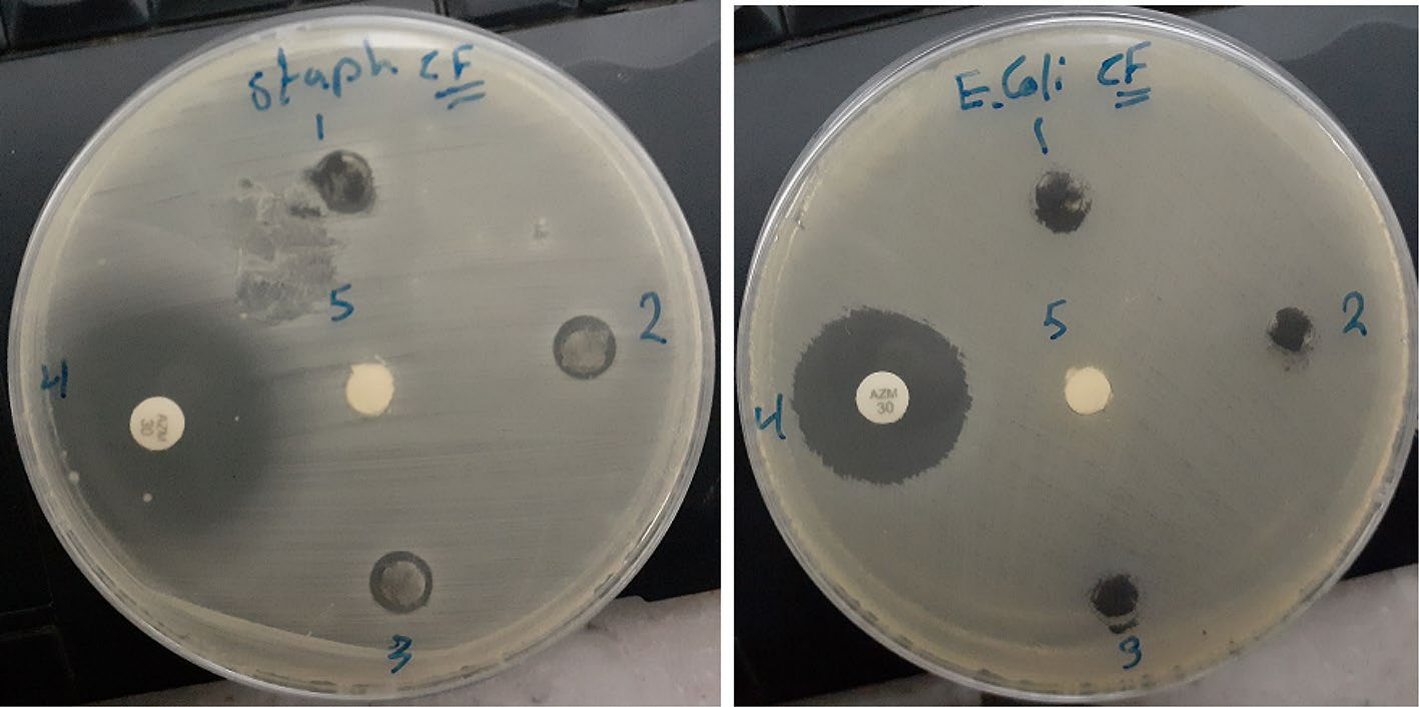
Antibacterial activity of Fe_2_O_3_–CuO–CuFe_2_O_4_ NCs against gram-negative bacteria (*Escherichia coli* and gram-positive bacteria (*Staphylococcus aureus*). (1) Fe_2_O_3_–CuO–CuFe_2_O_4_ NCs 50 mg/ml (2 Fe_2_O_3_–CuO–CuFe_2_O_4_ 100mg/ml (3) Fe_2_O_3_–CuO–CuFe_2_O_4_ NC 200mg/ml (4) Azithromycin (positive control) and (5) (d.H_2_O) (negative control).

**Figure 7:**
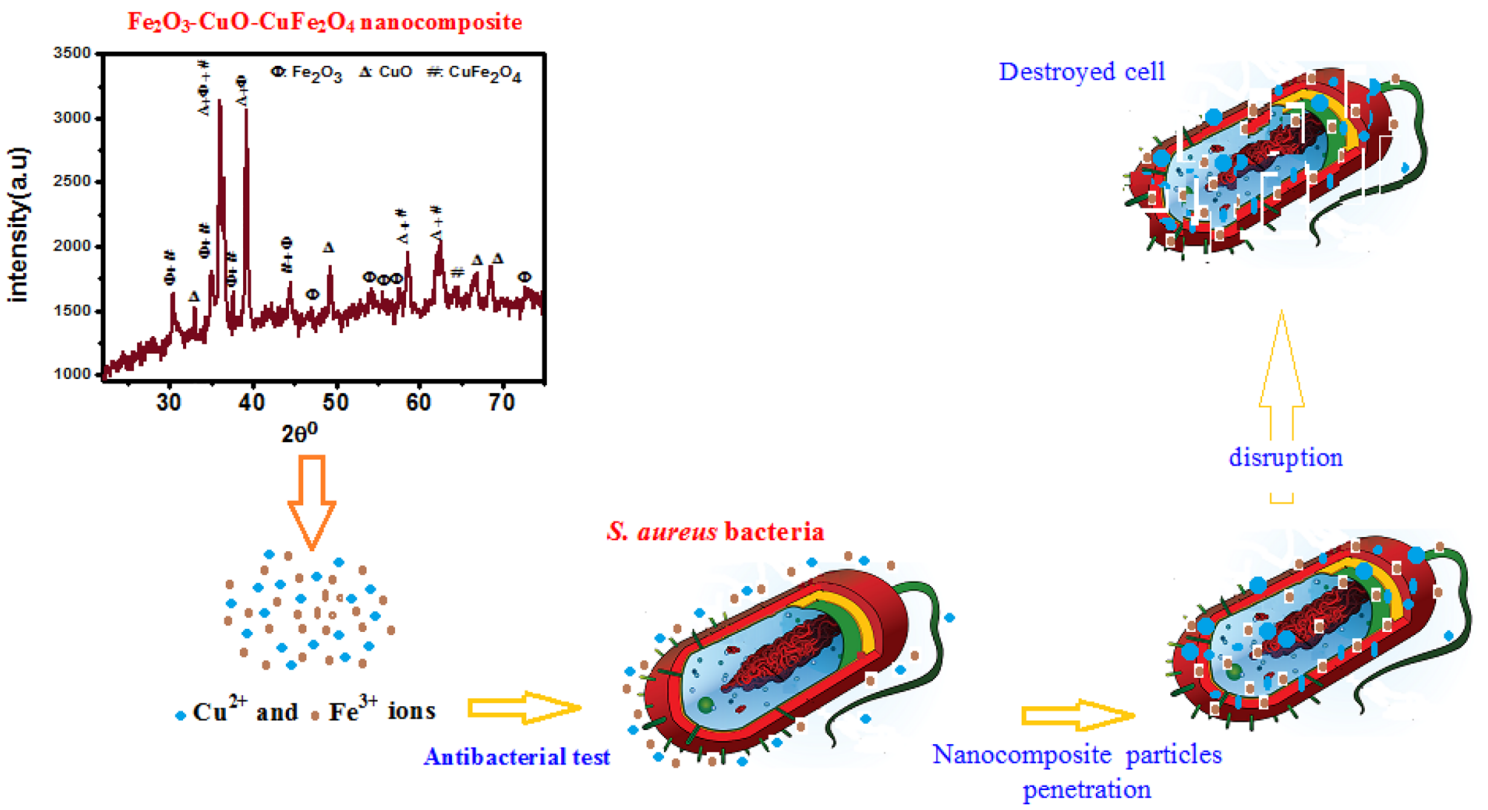
Proposed antibacterial mechanism of Fe_2_O_3_–CuO–CuFe_2_O_4_ nanocomposite.

The Fe_2_O_3_–CuO–CuFe_2_O_4_ nanocomposite was synthesized to assess its antibacterial efficacy against *Staphylococcus aureus* (gram-positive) and *Escherichia coli* (gram-negative). The results, as depicted in Fig. 6 and summarized in Table 3, revealed selective antibacterial activity. The nanocomposite effectively inhibited the growth of *S. aureus*, as indicated by a zone of inhibition (ZOI) diameter greater than 6 mm, which is considered to be a marker of potent antibacterial action. However, it failed to impede the growth of *E. coli*, where the ZOI diameter was 6 mm or less, denoting inadequate antibacterial activity.The selective action can be attributed to the structural differences between gram-positive and gram-negative bacteria. Gram-negative bacteria possess a less accessible, negatively charged peptidoglycan layer and a lipopolysaccharide-rich outer membrane that acts as a barrier to the nanocomposite’s active agents. Conversely, gram-positive bacteria are more vulnerable due to their thicker peptidoglycan layer which lacks such a protective barrier, enabling the nanocomposite’s active components to penetrate and exert their antibacterial effects^40,43,51^. The antibacterial properties of the Fe_2_O_3_–CuO–CuFe_2_O_4_ nanocomposite are primarily due to two mechanisms: the generation of reactive oxygen species (ROS) and the release of heavy metal ions. The Fenton reaction, facilitated by the nanocomposite under light irradiation, generates ROS, which induces oxidative stress leading to cellular damage. This stress results in intracellular disruptions, manifesting as DNA damage, lipid peroxidation, and protein oxidation, ultimately causing bacterial cell death^44,52–54^. Furthermore, the positively charged Cu^2+^ and Fe^3+^ ions from the nanocomposite interact electrostatically with the negatively charged bacterial cell wall, resulting in protein denaturation and disruption of DNA replication processes. These combined effects culminate in the inhibition of bacterial growth and cell death, elucidating the antibacterial mechanism of the Fe_2_O_3_–CuO-CuFe_2_O_4_ nanocomposite without adversely affecting nonbacterial cells^55–60^. A comparison of inhibition zone of the fabricated CuO–Fe_2_O_3_–CuFe_2_O_4_ nanocomposite with other multi-metal oxides from literature is given inTable4.To facilitate comparison, the test concentration and the method used were also tabulated. Figure 7 shows the mechanisms of the antibacterial activity of the nanocomposite. In the discussion of the results presented in Table 4, it is important to contextualize the observed Zone of Inhibition (ZOI) values within the framework of our specific experimental parameters. While it is acknowledged that the ZOI values observed in our study may be lower than those reported in other literature, it is imperative to consider the unique conditions under which our experiments were conducted. Factors such as the concentration of the nanocomposite, the incubation times, the specific bacterial strains used, and the methodology employed are all variables that can significantly impact the ZOI measurements. Moreover, it should not be automatically inferred that lower ZOI values equate to reduced antibacterial effectiveness. The diffusion rate of the nanocomposite within the agar, the interaction dynamics between the nanocomposite particles and the bacterial cell walls, as well as the potential for synergistic or antagonistic effects arising within the media, are all critical factors that need to be taken into account. These elements can considerably influence the antibacterial activity outcomes and may provide an explanation for the ZOI values obtained in our experiments. As such, the efficacy of the antibacterial properties of a substance should not be assessed solely based on ZOI values but should also consider the complex interplay of the aforementioned factors.

**Table 3:** Antibacterial activity of the Fe_2_O_3_–CuO–CuFe_2_O_4_ nanocomposite.

The prepared sample	Name of bacteria	ZOI (the diameter in mm) ± standard deviation(SD)			
		50 mg/mL	100 mg/mL	200 mg/mL	Control (Azithromycin)
Fe_2_O_3_–CuO–CuFe_2_O_4_	*S. aureus*	$7\pm 0.50$	$8\pm 0.25$	$9 \pm 0.75$	$27\pm 0.50$
	*E. coli*	–	–	–	29 $\pm 0.75$

**Table 4:** A comparison of antibacterial analysis of CuO–Fe_2_O_3_–CuFe_2_O_4_NC with some other nanocomposites.

Metal oxide nanocomposites (MO-NCs)	Test condition	ZOI (mm)		
		*S. aureus*	*E. coli*	Reference
CdO–NiO–ZnO	0.1 mg/mL, well method	–	16	^61^
CuO–MgO–ZnO	1000 mg/mL, well method	19	26	^1^
ZnO–Yb_2_O_3_–Pr_2_O	0.01 mg/mL, disk method	26	29	^8^
CdO–MgO	0.01 mg/mL, disk method	–	13	^18^
ZnO-CuO	0.01 mg/mL, disk method	12	6	^46^
Ag–Fe_2_O_3_	5000 mg/mL, well method	22	21	^62^
CuO–Fe_2_O_3_–MgO–CuFe_2_O_4_	100 mg/mL, disk method	19	12	^35^
gC_3_N_4_–BiFeO_3_–CuO_2_	0.15mg/mL, disk method	18	17	^63^
Fe_2_O_3_–Co_3_O_4_	20 mg/mL, disk method	22	23	^64^
CdS–ZnO	100 mg/mL, well method	17	11	^65^
ZnO–V_2_O_5_–WO_3_	0.003 mg/mL, disk method	18	–	^66^
CuO–Fe_2_O_3_–CuFe_2_O_4_	200 mg/mL, disk method	9	-	Present

The interplay between the optical characteristics of our Fe_2_O_3_–CuO–CuFe_2_O_4_ nanocomposite, namely the absorption edge at approximately 290 nm correlating to a bandgap of 4 eV, and its selective antibacterial efficacy against gram-positive bacteria, is a focal point of our study. This specific optical threshold implicates the nanocomposite’s proficiency in harnessing UV radiation to generate reactive oxygen species (ROS), potent agents of oxidation. These ROS are instrumental in attacking bacterial cell structures, particularly the less-protected peptidoglycan layers of gram-positive species such as *S. aureus*, resulting in notable cell damage and inhibition. We propose that the unique composition and structure of the nanocomposite promote a photocatalytic effect under UV illumination, which leads to the generation of ROS. These oxidative species are theorized to permeate the cell wall of gram-positive bacteria more readily than the more robust outer membrane of gram-negative bacteria, such as* E. coli*.

## Conclusion

The synthesis of Fe_2_O_3_–CuO–CuFe_2_O_4_ nanocomposite (NC) via sol–gel method was discussed. Ethanol was utilized as a capping agent to prepare NC. XRD, SEM, and UV–VIS Spectroscopy were employed to characterize the properties of the prepared material. XRD studies revealed that Fe_2_O_3_, CuO and CuFe_2_O_4_ exhibit cubic, monoclinic, and tetragonal phases respectively. UV–visible studies of the nanocomposite showed an energy bandgap of about 4eV. The nanocomposite exhibited antibacterial activity against gram-positive bacteria only. Based on the aforementioned evidence, Fe_2_O_3_–CuO–CuFe_2_O_4_ nanocomposite can be a suitable material for optoelectronic devices, diagnostic and therapeutic applications.

## Data Availability

The authors confirm that the data supporting the findings of this study are available within the article.
